# Are there Special Mechanisms of Involuntary Memory?

**DOI:** 10.1007/s13164-016-0326-z

**Published:** 2016-11-22

**Authors:** Christopher Mole

**Affiliations:** University of British Columbia, Department of Philosophy 1866 Main Mall, University of British Columbia, E370, Vancouver, BC V6T 1Z1 Canada

## Abstract

Following the precedent set by Dorthe Berntsen’s 2009 book, *Involuntary Autobiographical Memory,* this paper asks whether the mechanisms responsible for involuntarily recollected memories are distinct from those that are responsible for voluntarily recollected ones. Berntsen conjectures that these mechanisms are largely the same. Recent work has been thought to show that this is mistaken, but the argument from the recent results to the rejection of Berntsen’s position is problematic, partly because it depends on a philosophically contentious view of voluntariness. Berntsen herself shares this contentious view, but the defenders of her position can easily give it up. This paper explains how and why they should.

## Introduction

The past sometimes comes to mind when we do not intend to remember anything. For a number of reasons – some of them philosophical, some psychological, and some psychiatric – we should like to understand how these involuntary recollections differ from their voluntary counterparts. The most sustained attempt to address this question is Dorthe Berntsen’s 2009 book*, Involuntary Autobiographical Memory* (Berntsen 2009). Many psychologists have accepted the terms in which Berntsen poses her question, while rejecting the answer that she gives to it. The present paper takes a contrary stance: I claim that the position advocated by Berntsen is strong, but – owing to an inadequately-specified conception of the question – that position has been identified in a way that conceals its advantages.

Berntsen tells us that her work is intended to be “clearly psychological” (p. ix). Her contentions should therefore be thought of as hypotheses for empirical testing, rather than as candidates for philosophical proof. The present essay is, nonetheless, philosophical. It argues that Berntsen’s main contention can be formulated as an hypothesis that is strong and defensible; that it can be freed of problematic commitments; that it remains unthreatened by more recent discoveries; and that it has much to recommend it in the way of parsimony and plausibility. It is an empirical matter whether the hypothesis is true. Philosophical considerations cannot establish that, and the present essay does not purport to do so.

Involuntary recollections can have all sorts of emotional colourings. Recent studies have focused on those involuntary recollections that are unpleasant, and that are of unpleasant episodes (Clark et al. 2016). There are good reasons for this. Distressing recollections are central to the experience of post-traumatic stress disorder, and the recent research has often been motivated by the need to understand that condition, but it would be a mistake to suppose that the occurrence of involuntary memories is always experienced as unpleasant, or to suppose that it is essentially a symptom of disorder. When research has been conducted with older participants (in their mid-sixties and older) it has found that involuntary memories are frequently not rated as unpleasant (Schlagman et al. 2006). Many are reported as emotionally neutral. Distressing cases may be of the first clinical importance, but our theory of involuntary memories should allow them also to have a role in normal thought.

It is the attempt to understand involuntary memory as normal and healthy that gives the impetus to Berntsen’s enquiry. Her book begins by announcing its intention to “argue that although involuntary autobiographical memories may sometimes disturb us, we are generally lucky to have them” (p. 4); it concludes with the conjecture that involuntary memory makes “an important contribution to the flexibility of human behaviour” (p. 198). Berntsen clearly does not suppose that involuntary memories are experienced as unwelcome. What is it, then, in virtue of which these memories qualify as *involuntary?* The beginning of an answer is given on page two of Berntsen’s book when, after giving some positively valenced examples, she offers the following ‘operational definition’:

This book is about such involuntary autobiographical memories – operationally defined as memories that come to mind with no preceding conscious attempt at retrieval. They are contrasted with voluntary memories, which are memories that are called to mind in a strategic and goal directed fashion. (p. 2)

Immediately after this quotation, Berntsen stipulates that “These operational definitions do not imply that ‘free will’ is a causal factor for voluntary but not involuntary memories”, but even with this stipulation in place her definition skirts some philosophically contentious territories. If we insist on taking a route that passes through these territories then we will arrive at the debate that Berntsen is hoping to address only after becoming encumbered with philosophical baggage. The final section of this essay will return to some of the philosophical issues raised by Berntsen’s definition, but to avoid getting weighed down by them we should approach Berntsen’s position more directly.

## Berntsen’s Debate

There are any number of things that we do not know about involuntary memories, and any number of claims about them that might be debatable. Berntsen is concerned with a debate between what she calls the ‘Special Mechanisms View’ and what she calls the ‘Basic Mechanisms View’. According to the Special Mechanisms View there are mechanisms that are special to involuntary memory, in the sense of being mechanisms that are not also involved in voluntary memory. According to the Basic Mechanism View the mechanisms of involuntary memory are also mechanisms of voluntary memory. The maximal version of this Basic Mechanisms View would say that involuntary memory involves no mechanism that is not also involved in voluntary memory. (It should be noted that this would not imply that voluntary memory involves no mechanism that is not also involved in involuntary memory.)

Berntsen’s own sympathies are with the Basic Mechanisms View. She is particularly concerned to show that the explanation of involuntary memory does not require the postulation of such mechanisms as would be involved if involuntary memories were understood in Freudian terms, as being the result of a failure in some special mechanism of repression; a mechanism having no role in the voluntary case. Berntsen does not go so far as to assert the maximal version of the Basic Mechanisms View (although I will later suggest that she should). She does not rule out the existence of *some* special mechanisms. Her generalized suspicion of them is nonetheless made explicit when she writes that “Involuntary autobiographical memories […] are not so special that they should be explained in terms of their own memory system or in terms of memory mechanisms that pertain only to them” (p. 66).

The present essay is an attempt to vindicate this denial of specialness. A defence of it is called for, partly because Berntsen herself is not entirely consistent in maintaining it, and partly because recent experiments have suggested that involuntary memories may be special in ways that the Basic Mechanisms View fails to recognize. These experiments create problems for the position advocated in Berntsen’s book, as Berntsen herself formulates it, but I shall be suggesting that these problems originate with this formulation, and not with the substance of her claim.

Insofar as the problems facing Berntsen’s view are empirical ones, they have their crux in the question of *when* it is that the difference between involuntary and voluntary recollections gets made. In Berntsen’s own formulation the Basic Mechanisms View imposes a restriction as to when these difference makers occur. That restriction can be seen by reminding ourselves of the distinctions that psychologists have drawn between three processes that together contribute to the psychological career of a memory. The first of these is the process of storage, in which information comes to be encoded. The second is the process of consolidation, in which that information is reformatted in such a way that it becomes variously retrievable. The third is the process of retrieval, in which the information that has been laid down in a long-term store is brought back into consideration (Squire 1992; Tulving 1985).

A great many questions have been raised about this three-part taxonomy. It is not clear whether the occurrence of each process must manifest itself in consciousness (even if only in the penumbral consciousness of dreams (Stickgold 2005)). It is not obvious that the three processes must always take place in this order. (Perhaps the second can sometimes be skipped, or can be constituted by a reiteration of processes one and three (Meeter and Murre 2004)). And it is not clear whether the mechanisms responsible for these processes are psychologically independent of one another, so that each might be susceptible to its own distinctive pattern of breakdown (Alberini 2005). Although these questions are important, the controversies surrounding their answers should not cast doubt on the *reality* of all three processes. Nobody should doubt that memories are encoded, consolidated, and retrieved. The controversies concern how and when.

There should also be no question as to where, in this three-part taxonomy, we are to locate the *constitutive* difference between voluntary and involuntary memory. Although we have postponed the philosophically contentious business of giving a real definition of ‘voluntary’ and ‘involuntary’ memory, the essential difference between their referents is clearly a difference that gets made at the time when the memories are recollected. The question of whether my present recollections are happening voluntarily or involuntarily can only be a question about my psychological state *now*, at the time when these recollections are taking place. This moment of recollection is when the constitutive difference between voluntary and involuntary memory gets made*.* Our empirical questions concern the timing of whatever causal mechanisms bring about this constitutive difference.^1^

There is no reason to suppose that any of the three processes of memory is immune from malfunction, nor any obvious reason to suppose that the consequences of such a malfunction could not make some difference to the voluntary status of the recollections that result. On the face of it, then, any one of these processes might proceed differently in the voluntary and the involuntary case. This gives us three hypotheses to consider: (1) Involuntarily recalled memories might differ from voluntarily recalled memories in the processing by which they are first encoded; (2) they might differ in the processing by which they are consolidated; and (3) they might differ in the processing by which they are retrieved. These hypotheses are logically orthogonal, in that no one of them excludes any combination of the others.

In Berntsen’s own formulation of it, the ‘Basic Mechanisms View’ is presented as the conjecture that the last of these hypotheses is, by itself, sufficient to account for the various phenomena of involuntary memory, so that involuntary memories differ from voluntary ones *only* in respect of the processing by which they are retrieved. This interpretation is more or less explicit when Berntsen writes that “involuntary memories […] generally show the same characteristics as voluntary autobiographical memories with regard to factors related to encoding and maintenance” (p. 146). It is also indicated when she takes it to be incumbent upon her to show how the several differences between voluntary and involuntary memories:

can be explained as a byproduct of the dissimilar retrieval processes [and so] do not imply that the content of involuntary memories is encoded or maintained in ways that are fundamentally different from the encoding and maintenance of voluntary memories. (p. 67)

Psychology often proceeds via the conjecture, refutation, and refinement of bold hypotheses (as do other sciences, at various stages in their development). One might think that the results which have been established since the publication of Berntsen’s book have enabled progress of just this sort. Such progress would be understood as taking us away from the bold simplicity of the Basic Mechanisms View, by revealing that there *are* differences between voluntary and involuntary memories at the time of their encoding and maintenance. It is true that such differences have been observed. (We review some of them in the section that follows.) What is less clear is whether these findings reveal a flaw in Berntsen’s position, when the essential claims of that position are properly understood.

### Evidence for Differences at Encoding

When Berntsen’s Basic Mechanisms View is understood in the way suggested by the quotations just given – as saying that involuntary and voluntary memories differ only at the time of their retrieval – it is a view that can be threatened by evidence indicating that involuntary memories differ from voluntary ones at the time of their encoding or consolidation. The clearest evidence that the encoding of involuntary memories does indeed differ from the encoding of voluntary memories comes from a 2014 study, conducted by Clark et al. (Clark et al. 2014). In this study, as in several others relating to involuntary memory, participants watched a number of short films, depicting a series of somewhat distressing scenes. fMRI measurements were taken throughout the period during which these films were watched. The participants were then asked to keep a diary over the following week, in which they reported on which of the scenes they recollected involuntarily. (For a review of this method, see Holmes and Bourne 2008.)

These diary reports enabled the scenes that had been shown to be retrospectively classified, on a participant-by-participant basis, into one group containing those scenes that were subsequently remembered involuntarily, and one containing those that were not. The fMRI data from the original viewing of these scenes could then be analysed in order to see whether there was any difference between the initial processing of the two groups. The analysis showed that there was. The difference was subtle, but – by using Support Vector Machines that had been trained on the data from other participants^2^ – it was possible to distinguish with some accuracy between those patterns of neural activation that had been elicited during the initial viewing of scenes that were remembered involuntarily, and those that had been elicited during the initial viewing of scenes that were not so remembered. The authors of this study suggest that the difference that their Support Vector Machines pick up on corresponds to a contrast that is specific to the way in which to-be-involuntary memories get encoded.

Whatever the correct interpretation of the contrast that Clark et al. observe, the existence of that contrast does gives evidence that the initial processing of involuntary memories differs in some way from the initial processing of voluntary ones. It does not, by itself, tell us very much about the psychological character of that difference, but we do not need a theory of that psychological character in order to see that the results of this experiment weigh against Berntsen’s suggestion that involuntary and voluntary memories differ ‘simply with respect to the way they come to mind’. Such memories seem also to differ, in some way, in the processing that they receive at the time of their first encoding.

### Evidence for Differences at Consolidation

The process of consolidation is somewhat harder to study than the process of initial encoding. It happens over a longer and more flexible timescale, and therefore does not lend itself to observation in a brain scanner. (Attempts to study it in that way, one of which we shall be considering in a later section of this paper, have nonetheless been somewhat successful.) Despite these methodological difficulties, there is evidence suggesting that, during the process of consolidation, involuntary memories are handled in a way that contributes to their involuntariness.

Such evidence comes from studies in which interventions that are thought to disrupt the process of consolidation are shown to influence the likelihood of intrusive recollection. One consolidation-disrupting intervention is the prevention of sleep. Porcheret et al. (2015) found that participants who slept as normal after watching traumatic films were significantly more likely to recollect those films involuntarily than were participants who caused themselves to remain awake for the following night (Porcheret et al. 2015). This suggests (although it does not, by itself, establish) that some of the consolidatory processing that takes place during normal sleep contributes to the occurrence of involuntary recollections.

Sleep is not the only occasion on which memories are consolidated. The cognitive resources that are involved in waking consolidation are believed to overlap with those that are involved in visual-spatial working memory (Ranganath et al. 2005). It is therefore plausible that consolidation might be disrupted by placing a high load on this form of working memory, thereby making its resources unavailable for use in the service of consolidation. Video games often place a high load on visual-spatial working memory. Holmes et al. (2009) therefore instructed some of their experimental participants to play Tetris for ten minutes, half an hour after having watched a series of traumatic films. They found that these Tetris-playing participants were significantly less likely to have involuntary recollections of the films than were participants who had not played any video game. Involuntary recollections did still occur, but they occurred with a significantly lower frequency (Holmes et al. 2009). A subsequent study found that participants who played Tetris were also less likely to have involuntary recollections than were participants who played a game without a visual/spatial component (Holmes et al. 2010). This again suggests that consolidatory processing contributes to the occurrence of involuntary recollections, by showing that the disruption of such processing reduces the likelihood of involuntary recollections occurring.

The playing of Tetris and the prevention of sleep might have several cognitive effects. The results of these experiments are therefore susceptible to different interpretations, but in each case it seems likely that an intervention taking place some time after the initial encoding of an episode, and some time before that episode is recollected, can influence the involuntariness of that subsequent recollection. Since this is the characteristic time window for processes of consolidation, these experiments suggest that differences taking place during the process of consolidation can bring about differences of voluntariness. Again this appears to weigh against Berntsen’s suggestion that involuntary and voluntary memories differ ‘simply with respect to the way they come to mind’. Something happening during the process of consolidation seems also to make a causal contribution to involuntary recollections.

## Defending the Basic Mechanisms View

The previous sections have considered differences between involuntary and voluntary memories at the time of their encoding, and at the time of their consolidation. This leaves us to consider the differences between voluntary and involuntary memories at the time of their retrieval. Observations of such differences have been made by Hall et al., using positron emission tomography (Hall et al. 2008). Nobody should be surprised by the fact that such differences exist. These are, as we have said, constitutive of the contrast between voluntariness and involuntariness. Berntsen’s question is whether these differences at the time of retrieval are the only differences that matter, for the purposes of explaining why it is that involuntary recall occurs. Her version of the Basic Mechanisms View suggests that they are. The research that we reviewed above suggests that it is wrong to do so. This has led current researchers to focus on the ways in which information about involuntarily remembered events is encoded and consolidated (Segovia et al. 2016; Clark et al. 2016; Spring et al. 2015). The results of this research are undeniably valuable, but I want to suggest that the abandonment of Berntsen’s Basic Mechanisms View gives up an important insight, and that the adoption of its rival – the ‘Special Mechanisms View’ – would be a mistake.

Berntsen formulates the Basic Mechanisms View in various ways. In some formulations, that view seems to include the idea that the only differences between voluntary and involuntary memories are differences that occur at the point when they come to mind (p. 67), and so the view seems to be committed to saying that these memories do not differ from each other at the time of their encoding and maintenance (e.g. p. 84). These are claims that the evidence reviewed above shows to be mistaken. It is this that creates the impressions that what we have here is a straightforward case of the scientific method proceeding via the refutation of a bold conjecture that had initially seemed like it might be adequate to the phenomena.

We should remember, however, that the debate between the Basic Mechanisms View and the Special Mechanisms View was intended to be a debate about the existence of *mechanisms* and of *systems*. In rejecting the Special Mechanisms View, Berntsen’s claim is that “involuntary autobiographical memories […] are not so special that they should be explained in terms of their own memory system or in terms of memory mechanisms that pertain only to them” (p. 66). Not every difference in the brain is a difference of mechanism, and so the discovery of some differences relating to encoding and maintenance does not suffice to show that different mechanisms of encoding and maintenance must be at work in the voluntary and involuntary cases. The question of whether we should take the step to that last claim depends on when it is that two bits of neural activity are different enough to qualify as instantiating different mechanisms. Our answer to this is partly a matter of verbal stipulation, but the point is more than a merely verbal one. Its substance can be seen if we compare the differences between voluntary and involuntary memories with differences that have been observed, by similar means, in quite different experimental contexts.

The first line of evidence that we considered above showed that there are differences in the patterns of neural activation that are elicited by a scene, at the time of its encoding, depending on whether that scene will or will not turn out to be involuntarily remembered over the course of the following week. The differences between these patterns of activation were not large. The evidence for their existence was that Support Vector Machines can be trained to classify sets of fMRI data on the basis of them. We should not suppose that, if a difference at the time of encoding can be picked up on by Support Vector Machines, then that difference is evidence of different encoding mechanisms being at work. To suppose this would be to find ourselves postulating special mechanisms with all sorts of spurious purposes.

This can be illustrated by considering a 2012 experiment, in which Polyn et al. presented their participants with a series of to-be-remembered words, referring to commonplace items (Polyn et al. 2012). In some contexts the participants were given the task of judging whether these items were animate or inanimate. In others they were given the task of judging whether those same items could be fitted into a shoe box. Support Vector Machines were able to discriminate between those patterns of activation elicited by items that were being encoded during the animacy task and those that were elicited by items that were being encoded during the size task. There must be some difference that these Support Vector Machines are successfully picking up, but nobody would take the existence of such a difference as indicating that there are special mechanisms for the encoding of information pertaining to items whose animacy one has judged. The basic mechanisms for encoding memories about things that one has judged to be larger than a shoebox are still quite plausibly the same as the basic mechanisms for encoding memories about things that one has judged to be inanimate. A defender of Berntsen’s Basic Mechanisms View should make the same claim about the contrast between voluntary and involuntary memory: the basic mechanisms for encoding memories of things that one will remember involuntarily can still quite plausibly be the same as the mechanisms for encoding memories of things that one will remember voluntarily, even if the events that take place during these encodings are different enough to enable accurate classification by a Support Vector Machine.

A similar point can be made about differences that are observed at the time of consolidation. It has been shown that patterns of activation during the period of consolidation differ, depending on what it is that the subject has been asked to remember, in ways that Support Vector Machines can discriminate. We noted above that the period of consolidation is exceptionally difficult to study (since consolidation can take place at various times, and especially during sleep). These difficulties were faced head-on in a 2013 study by Deuker et al., in which participants were given the task, in a brain scanner, of memorizing associations between locations on a screen and various arbitrary items, including keys, cars, hamsters, and pictures of Angela Merkel (Deuker et al. 2013). The participants then returned to the scanner for a couple of hours of rest, so that the consolidation of these memories could be observed. The various items needing to be remembered in this experiment were grouped into two sets. One of the several interesting things that the data from this experiment show is that Support Vector Machines are able to discriminate between the activity elicited during the consolidation of items in Set A and the activity elicited during the consolidation of items in Set B. Both sets contained a mix of animate and inanimate items, familiar and unfamiliar ones, large ones and small ones. The division of items between the two sets was quite arbitrary. It is therefore overwhelmingly likely that the basic mechanisms for the consolidation of memories about things in the first set were the same as the basic mechanisms for consolidating memories about things in the second. This claim is in no way threatened by the fact there is *some* difference – discernible with the help of Support Vector Machines – between the behaviour of those mechanisms during the consolidation of memories for items in set A, and the behaviour of those mechanisms during the consolidation of memories for items in set B (nor has anyone ever supposed that it is). The point here, as before, is that the existence of a discernable difference does not, by itself, entail a difference of *mechanism*, least of all when it is the sort of difference that can be discerned only with the aid of a powerful learning algorithm, such as a Support Vector Machine.

In the absence of evidence for a difference of *mechanism*, the Basic Mechanisms View will remain plausible. In order to threaten the plausibility of that view our evidence would need to show, not only that something relevant to involuntariness is going on during the encoding and consolidation of involuntary memories, but that it is going on in a way that requires a distinct mechanism for its explanation. The discovery that involuntary memories can be made less likely by playing Tetris, or that they can be made less likely by the interruption of sleep, establishes only the weaker of these. Those interventions might influence involuntary memory by influencing some one basic memory mechanism, by the action of which voluntary and involuntary memories are both consolidated. There are therefore interpretations of the Basic Mechanisms View with which such discoveries are compatible. The following section brings one such interpretation into view, as being the interpretation that a defender of Berntsen ought to favour.

## An Alternative Conception of the Debate

Rather than conceiving of our central debate as being concerned with a question about the locus of difference-makers, we can instead understand the contrast between Berntsen’s Special and Basic Mechanism theories by thinking of it on the model of older debates that were concerned with the distinctness of memory systems. There was a time when such debates were all the rage, and when great care was taken to consider what would be required in order to show that the distinctions between implicit and explicit memory, or between declarative and procedural memory, were to be explained by reference to a difference in the *systems* underpinning them (Cohen and Squire 1980; Rugg et al. 1998).

Those debates had their roots in some basic issues concerning cognitive architecture, which can be brought to light by reflecting on those situations in which the brain seems to be representing the same body of information twice over. An example is given by the information that is required to correctly parse a sentence in one’s first language. One’s brain must have had *some* representation of that information for at least as long as one has been able to speak grammatically. When, much later in life, one comes to think in a philosophical or linguistic way about the conditions on grammaticality, one’s brain seems to need an altogether new representation of the same information. It is therefore plausible that there are two separate ways in which information about the grammar of a language can be stored. If we suppose that any psychologically-useful information storage deserves the name of ‘memory’ then we should find it plausible that, in the brain of any speaker who is both fluent and linguistically-knowledgeable, there are two separate memory systems, both handling information about the grammar of that speaker’s language. Although it is not innocent of complications, the empirical investigation of this hypothesis faces relatively few philosophical pitfalls. It can proceed by the empirical examination of different patterns in learning; of different patterns in breakdown and interference; and of different patterns in neural activations elicited by various tasks (Evans 1985, pp. 331–333).

The question of whether different information-storage phenomena require different systems of memory for their explanation is one that can be asked in a broad variety of contexts. Berntsen’s debate between the Basic Mechanisms View and the Special Mechanisms View can be understood as one version of it. On this understanding, the Special Mechanisms View treats the distinction between voluntary and involuntary memory as being somewhat analogous to the distinction between implicit and explicit memory of grammatical rules. It says that voluntary and involuntary memory need separate memory systems for their explanation, whereas the Basic Mechanisms View says that there is no such need, and that the same systems – operating in somewhat different ways – can explain both sets of phenomena.

Considerations of prima facie plausibility clearly speak in favour of the Basic Mechanisms View, on this conception of it. A memorable episode from the past might intrude unexpectedly into the stream of consciousness on one occasion. On some other occasion that same episode might be recalled quite voluntarily, and incorporated into the stream of consciousness as a result. The same body of information would seem to be at work in both the voluntary and the involuntary case: in each case this would be information about the experience of some one episode in the past; in each case such information would manifest itself by coming to be present in consciousness, and thereby coming to be available for verbal report, and for processing by the various forms of working memory. The contrast between voluntary and involuntary memory is, in these respects, quite unlike the contrast between our tacit and explicit knowledge of grammar. The considerations that motivate the postulation of distinct memory systems in the case of language find no application here. And so, contrary to the verdict suggested by our earlier interpretation, it now seems quite natural to *agree* with Berntsen’s suggestion that “involuntary autobiographical memories differ from their voluntary counterparts simply with respect to the way they come to mind” (p. 67).

If we interpret it in this way then the Basic Mechanisms View is also supported by general considerations of parsimony. One of the lessons to be learned from research into the psychology of memory in the twentieth century was that distinctions between different memory systems have a tendency to proliferate (Schacter et al. 2000). Opportunities to distinguish between memory systems should therefore be regarded with caution, since they threaten to result in the fragmentation of the bigger and more explanatory picture. In asking whether the phenomena of voluntary and involuntary memory require different systems for their explanation, we should be wary of supposing that any psychological difference between these varieties of memory must correspond to a difference as to the psychological mechanisms underpinning them. When Berntsen rejects the Special Mechanisms View of involuntary memory – and tells us that “Involuntary autobiographical memories […] are not so special that they should be explained in terms of their own memory system or in terms of memory mechanisms that pertain only to them” (p. 66) – these considerations of parsimony speak in favour of her position.

On the interpretation suggested by the earlier debates about memory systems, the Basic Mechanisms View ceases to look like a bold opening conjecture, refuted by more recent work. It starts to look like a sensible starting position, to be abandoned only if the evidence requires it. The sort of evidence that would require us to abandon that view would be evidence of different *mechanisms* being at work. Such evidence cannot be provided by the use of Support Vector Machines to uncover subtle differences, nor by the discovery of interventions during periods of consolidation that reduce the incidence of involuntary recollections. To the extent that these methods reveal differences, they need not be differences of mechanism.

## The Maximal Basic Mechanisms View

On the interpretation that we have just offered the Basic Mechanisms View looks so plausible that even its maximal version – which says that involuntary memory involves no mechanism that is not also involved in voluntary memory – would seem to be defensible. This maximal view is not Berntsen’s, since she is willing to allow that the two varieties of memory do involve different mechanisms at the point of retrieval, provided that these are “different retrieval mechanisms operating on the same episodic memory system.” (p. 67). I want to suggest that we can resist this concession, and can deny that involuntary memories involve any special mechanisms of retrieval, just as we have denied that they involve any special mechanisms of encoding and consolidation. Hitherto, our tactic for the defense of the Basic Mechanisms View has been to follow Berntsen in saying that involuntary memories “are special in some regards […] However, they are not so special that they should be explained in terms of their own memory system or in terms of memory mechanisms that pertain only to them” (p. 66). In deploying this tactic we have been admitting that there is some difference between the voluntary and the involuntary case, while denying that these differences require any difference of mechanism for their explanation. If we attempt to redeploy that tactic here, when considering differences at the time of retrieval, we face some informative complications.

We have seen that everybody needs to admit that involuntary memories differ from voluntary ones at the time of their retrieval, since some such difference is constitutive of the contrast in which we are interested: the retrieval of involuntary memories *must* be different from the retrieval of voluntary ones, for otherwise they would cease to qualify as involuntary. Their involuntariness consists in their being retrieved in an involuntary way. A defender of the maximal Basic Mechanisms View therefore needs to claim that even this constitutive difference is not a difference of *mechanism*. I suspect that Berntsen’s adoption of a less-than-maximal view is motivated by her thinking that this last claim would not be tenable: her position seems to be that the differences between voluntary and involuntary memories at the time of their retrieval *must* be differences of mechanism. But I suspect that Berntsen believes this last claim for reasons that are derived from her philosophically contentious conception of voluntariness. In order to see how that claim can be resisted, we shall need to consider this conception, and so will need to return to the philosophically contentious ingredients in Berntsen’s operational definition, which were set aside in this essay’s opening section.

In our attempts to interpret Berntsen’s position, we have repeatedly quoted her claim that “the essence” of the Basic Mechanisms View is “that involuntary autobiographical memories differ from their voluntary counterparts simply with respect to the way they come to mind.” From here Berntsen immediately goes on to say that “The main difference in this regard is that involuntary memories are activated with no preceding goal-directed search and with no search description” (p. 67). We can explicate the conception of voluntariness that is implied by these remarks – and by the ‘operational definition’ that was quoted earlier – if we cancel out the negations. Doing so shows Berntsen to be taking it that *voluntary* memories are activated *with* a ‘preceding goal-directed search’ or ‘search description’. In order see why this should be taken as requiring that a different *mechanism* must be at work in the involuntary case, it may help to state the point in somewhat different jargon.

If we frame the point in the terms that David Marr introduced, in his agenda-setting work on vision (Marr 1982), then we shall say that, in the case of voluntary memory, Berntsen is taking it that the mechanisms of recall compute some function that takes ‘search descriptions’ as an argument (together with contexts) and that returns some particular memory as its value, whereas in the case of *involuntary* memory, she is taking it that the mechanisms of recall must be computing some function that has no search description among its arguments. Since they have different inputs, and different adicities, these must be different functions. They must therefore require different algorithms for their computation. In a project that aims to occupy a level of description that is ‘clearly psychological’, rather than being philosophical or neurological, the operation of different algorithms can plausibly be taken as a sufficient criterion for there being a difference of mechanism. And this would make the maximal version of the Basic Mechanisms View appear to be untenable: cast in Marrian terms, Berntsen’s thought seems to be that different functions require different mechanisms for their computation, and that the function computed in voluntary memory must be different from the function computed in the involuntary case, since only one of these has an argument place for search descriptions.

Whatever its merits, the preceding line of thought cannot get going if we reject the conception of voluntariness from which it starts. The weaknesses of this conception can be seen, without the need for too much philosophical machinery, via the use of some counterexamples. Such examples suggest both that there can be involuntary memories that *are* retrieved in response to a consciously-entertained search description, and that there can be voluntary memories that are not. They therefore suggest that computing a function that takes search-descriptions as an input is not distinctive of the voluntary case.

For an example of the first sort suppose that you are, quite voluntarily, reminiscing with friends about your school days. The memories that you are recollecting are being retrieved on account of the fact that you are searching for school-related memories. If you had not been searching for them, you would be thinking about something entirely different. Even in a context such as this you might find yourself assailed by memories from some particular traumatic episode, in which some sadistic schoolmaster picked you as his victim. If such memories are intrusive, inevitable (given the context), and unexpected – and if, despite your best efforts, they persist once they have been recalled – then they can perfectly well be experienced as involuntary, even if the conscious entertainment of a search description was the cause of their occurrence. There is nothing fanciful about examples of this sort. The post-traumatic flashbacks that give one paradigm case of involuntary memory can occur with the loss of control that gives them their involuntary character, even when their occurrence is prompted – as it might be in a therapeutic context – by a conscious search that is deliberately undertaken. An instance of memory retrieval can therefore be experienced as involuntary, even if the processing by which it is caused takes, as a computational input, the conscious entertainment of an intentional search term.

For a counterexample of the opposite sort, in which voluntariness requires the input of no such consciously entertained search term, suppose that you have asked me to buy bread on my way home from work. I recollect your asking me to do so as I am passing the bakery, and buy the bread accordingly. The case is one in which I do what I had intended, in the intended way, and for the intended reason. Nothing involuntary is going on. It would therefore seem to be a mistake to suppose that my recollection of your asking needs to be prompted by ‘a conscious attempt at retrieval’ in order for it to be voluntary. That recollection *may* be prompted by an attempt at retrieval, but only if I have *already* remembered that there is something I need to buy on the way home. There is no need to postulate such an attempt in every case, and it would be to embark on a regress to suppose that voluntariness always requires that a prior attempt be voluntarily made. The voluntary recollection of your asking me to buy bread can perfectly well be initiated, not by the conscious entertainment of a search description, but by the smell coming from the bakery.

Episodes of this second sort qualify as being *voluntary* because the subject of the recollection is *in control* of the larger-scale conduct to which that recollection contributes, and of which it is a part. Episodes of the first sort qualify as *involuntary* because the subject is *not* in control. These examples work because they are cases in which control is lost, or is taken, after the recollection is initiated. They therefore weigh against Berntsen’s contention that “The main difference” between voluntary and involuntary memories “is that involuntary memories are activated with no preceding goal-directed search and with no search description” (p. 67): Rather than being thought of as a difference as to how they are *activated*, this ‘main difference’ should instead be thought of – in the style of Frankfurt 1978 (p. 157) – as a difference as to how the mental episodes to which they contribute are controlled, over the course of their temporal development. This removes our reason for thinking that the computation of a different algorithm must be responsible for the recollection of the voluntary and involuntary cases. It therefore removes the obstacle to maintaining a maximal form of the Basic Mechanism View, according to which involuntary memory involves no mechanism that is not also involved in voluntary memory.

The truth of this view would be a matter of some significance, and the premature rejection of it could skew the agenda of our research. Some of the current research into involuntary memory construes itself as taking ‘key steps’ towards ‘developing a cognitive vaccine against traumatic flashbacks’ (Holmes et al. 2010). If the maximal Basic Mechanisms View is true then every mechanism that contributes to involuntary memory also contributes to voluntary memory. The plausibility of that view reduces the likelihood of there being any clinical intervention that directly and selectively acts against involuntary memory, while leaving voluntary memory intact, since the view entails that there is no mechanism at which this intervention could be directed. The argument for thinking that it should be possible to develop ‘a cognitive vaccine against traumatic flashbacks’ is therefore more controversial than might have been supposed.

## Footnotes

1 There is a quite different sense of ‘involuntary memory’, according to which that term refers to the involuntarily *storage* of information, rather than the involuntary *recollection* of information, but a study of the difference between voluntary and involuntary storage would use entirely different methods from Berntsen’s. It is not that sense of ‘involuntary memory’ that is being employed in the present discussion.

2 Support Vector Machines are one variety of machine learning algorithm. Given a set of complex patterns that have been classified as belonging to various categories, these algorithms are able to learn how new patterns should be categorized. In the experiment under discussion, Support Vector Machines are trained on fMRI data from participants who are watching traumatizing films. The machines learn to classify these into one group, containing data from cases in which the film would be involuntarily recollected, and another group, containing data from cases that would not. Support Vector Machines do not require the members of each category to be clustered together in a circumscribed region of the feature space in which they are represented. Instead they are able to project that feature-space into a new representational space, with much higher-dimensionality. The clusters that define each category need only be circumscribable within these much-higher dimensional representations. This enables Support Vector Machines to efficiently learn categories by which other learning algorithms are defeated.
